# Induction of Anti-Proliferative and Apoptotic Effects of Sorafenib Using miR-27a Inhibitor in Hepatocellular Carcinoma Cell Lines

**DOI:** 10.31557/APJCP.2021.22.9.2951

**Published:** 2021-09

**Authors:** Abdel Hady A Abdel Wahab, Eman G Ayad, Mohga S Abdulla, Hayat M Sharada, Abeer M Ashmawy

**Affiliations:** 1 *Department of Cancer Biology, National Cancer Institute, Cairo University, Egypt. *; 2 *Department of Chemistry, Faculty of Science, Helwan University, Egypt. *

**Keywords:** Sorafenib, HCC, miRNAs, miR-27a, HepG2 cells, Huh7 cells

## Abstract

**Objective:**

The purpose of the current study was to investigate the possible anti-tumor effect of miR-27a inhibitor in combination with Sorafenib (SOR) on cell proliferation and apoptosis of hepatocellular carcinoma cell lines.

**Methods:**

Transient transfection by oligo-miR27a inhibitor (miR-27ai) was used in this study for targeting the oncogenic miR-27a in HepG2 and Huh7 cells followed by SOR treatment. Cell viability was measured using SRB assay. The cell cycle and apoptosis were assessed by flow cytometry assay. Moreover, the level of oncogenic miR-27a was evaluated in 19 tissues of primary HCC patients as well as cell lines using qRT-PCR assay. Finally, caspase-3 activity was determined using ELISA assay.

**Results:**

Significant up-regulation of miR-27a expression was reported in HCC patients confirming its oncogenic role. Treatment of cells with SOR following transfection with miR-27ai declined cell viability significantly compared with either control or single agent treatment (p≤0.05). Highly significant decreasing in the number of cell in S-phase associated with increasing in G0-phase was also observed. Furthermore, apoptotic rate was highly significantly increased for transfected/SOR treated cells (p≤0.01). Finally, combination treatment demonstrated a significant elevation of caspase-3 activity level in both cell lines examined.

**Conclusion:**

The present data demonstrated targeting miR-27a enhances the anti-tumor effect of SOR in HCC cell lines considering as one of the promising therapeutic targets for advanced HCC management.

## Introduction

Hepatocellular carcinoma (HCC) is considered as one of the most commonly diagnosed malignancies and the third-leading cause of cancer-related death in the world after lung and stomach. HCC incidence mainly associated with chronic hepatitis B virus (HBV) and hepatitis C virus (HCV) infections (Ghouri et al., 2017). HCC has a very low overall 5-year survival rate due to lake of definite and accurate diagnostic tools in its early stages (Golabi et al., 2017). Low survival benefits were recorded for late-stage HCC patients received systemic chemotherapeutic agents (Liu et al., 2015). Sorafenib (Nexavar) is a multikinase inhibitor that targets both RAF and a number of tyrosine kinases, including vascular endothelial growth factor receptors (VEGFRs), and platelet-derived growth factor receptor- β (PDGFR-β) (Wilhelm et al., 2005). Despite the fact that sorafenib has been approved in several countries worldwide for advanced HCC patients still yet the drug show unsatisfied clinical outcomes due to its severe side effects as well as drugresistanceleadingto tumor progression and recurrence (van Malenstein et al., 2013). Furthermore, resistance to treatment, tumor recurrence and metastasis shed the light of the importance of providing novel effective therapeutic tactics able to enhance the clinical outcome for HCC patients. 

MicroRNAs (miRNAs) are a group of noncoding RNAs that are very conservative with small sequence of nucleotides (17-25nt) which play important regulatory roles in translation of target messenger RNAs through base pairing with the 3′ untranslated region (3′UTR) of their target genes resulting in either degradation or translational inhibition of those genes (Lewis et al., 2005). Growing evidences were accumulated in recent decades emphasizing the crucial role of these small non coding RNAs in regulation of many target genes related to a lot of cellular processes such as cell proliferation, development, differentiation, and apoptosis. Additionally, numerous studies linked between deregulated miRNAs and cancers including HCC where these miRNAs regulate many of the aggressive traits of the disease including tumor progression, metastasis and drug resistance (Zhang et al., 2017; Li et al., 2017).

Interestingly, various studies spotted lights on changes in miRNA expression profiles in many cancer types in response to much therapeutics including sorafenib. It has been shown that miR-26a plays a crucial function for inhibiting angiogenesis and metastasis via targeting VEGFA and HGF. Its low expression was reported in metastatic HCC tissues compared with their corresponding normal referring to its possible therapeutic target against metastasis (Yang et al., 2014). Another study showed an enhancement of sensitivity of HCC cell lines to doxorubicin and vincristine treatment upon upregulating miR-122 expression level through suppressing Bcl-w and cyclin D1 protein levels that are involved (Xu et al., 2011). 

miR-27a, one of the miRNA-27 family, was located on chromosome 19. Growing evidence has confirmed its vital role in regulating various biological processes in human cells including proliferation, migration, invasion and metastasis.Furthermore, its clinical significance as a therapeutic agent of cancer and drug sensitivity. It has been demonstrated that miR-27a was significantly overexpressed in different type of tumors including HCC refereeing its oncogenic property (Li et al., 2015). Meanwhile, it behaves as a tumor suppressor in other types of tumor (Bao et al., 2014). Previous study demonstrated that suppression of miR-27a level sensitized colorectal stem cells to TRAIL (Zhang et al., 2017). 

Therefore, the aim of the present study was to explore the anti-tumor effect of miR-27a inhibitor in combination with sorafenib treatment on HCC cell lines (HepG2/Huh7) that might provide a useful strategy for enhancing hepatocellular carcinoma treatment.

## Materials and Methods

*Materials*

Dulbecco’s modified Eagle’s medium (DMEM), fetal bovine serum (FBS), Penicillin and streptomycin were obtained from (Sigma-Aldrich Chemical Co., St. Louis, MO, USA). Sorafenib was purchased from the Bayer Corporation (West Haven, CT). For a 10 mM stock, the 10 mg reconstituted in 1.57 ml DMSO. The final concentration of DMSO in medium was 0.1% (v/v).

*Cell lines and tissue specimens*

Human hepatocellular carcinoma cell lines HepG2 and Huh7 were purchased from VACSERA (The National Holding Company for Biologics and Vaccines, Cairo, Egypt). The two cell lines were cultured in Dulbecco’s Modified Eagle’s Medium (DMEM) supplemented with 10% fetal bovine serum (FBS) and 1% penicillin/streptomycin then incubated at 37°C in a highly humidified atmosphere incubator with 5% (v/v) CO_2_ and 95% air. The cell lines regularly sub-cultured to be in there exponential growth rate.

Nineteen formalin fixed paraffin embedded (FFPE) specimens were obtained from newly diagnosed primary HCC patients at the Egyptian National Cancer Institute, Cairo University. Five FFPE specimens were collected from healthy liver donors’ prior transplantation as controls. A written informed consent was obtained from the individual included in the study and the study was approved by the Institutional Review Board of the Egyptian National Cancer Institute, Cairo University. 

*Cell proliferation assay*

Sulphorhodamine-B (SRB) assay (Sigma-Aldrich Chemical Co., USA) was conducted for assessing the cytotoxic effect of Sorafenib on the growth of HuH-7 and HepG2 cells. Cells were seeded in plate 96-multiwell plate (1X10^4^ cells/well) in fresh medium and cultured for 24 hrs treatment until reaching 50–60% confluence, then treated with different concentrations of Sorafenib (3, 4, 8, 16 and 32µM ) for 48 hrs to determine the most effective dose to be combined with miR-27ai.Following treatment, cells were fixed with 50 µl cold 50% TCA for 1 h at 4°C. Wells were washed once with distilled water and stained for 30 min at room temperature with 50 µl 0.4% SRB dissolved in 1% acetic acid for 10 min. Unbound SRB was removed by washing five times with 1% acetic acid. After dried, protein-bound dye was extracted with 100 µ1 of 10 mM Trisbase (pH 10.5). Control cells were treated with 0.1% DMSO alone and the absorbance was measured at 570 nm using a microplate reader (Tecan SunriseTM, Germany). Each treatment was done in three independent experiments.

*Anti-miR-27a transfection*

HepG2 and Huh7 were incubated at 37°C in a 96-well plate (1X 10^4^ cells/well). Just prior to transfection, the media were replaced with 50μl serum free media. MiR-27a inhibitor and miR-NC (purchased from Qiagen, Germany) were transfected into the cells using HiPerFect transfection reagents according to the manufacturer’s instruction.After transfection, cells were treated with sub-IC_50 _doses of sorafenib (IC_20_ and IC_35_) for 48 hrs. The cells were performed for cell viability, apoptosis, cell cycle, qRT-PCR and caspase-3 assay.

*Quantitative RT-PCR*

Total RNA from cell lines and FFPE sections was purified using miRNeasy Mini and miRNeasy FFPE kits (Qiagen, Germany) following manufacture instructions. cDNA was synthesized from 1 ug of RNA using miScript RT II kit (Qiagen) according to the supplied protocol.The expression level of miR-27a was determined using SYBER Green reagent kit (Qiagen). About 5 ng of cDNA was used as template in a 10 μl PCR reaction containing 1X SYBR Green master mix, 1X miRNA specific forward primer, and 1X universal primer. All the RT-qPCR reactions were performed on ViiA7 real-time PCR system (Applied Biosystems, USA). All samples were performed in triplicate. U6 snRNA was used as an endogenous control for normalization. Data were expressed as fold change, using the 2^-∆∆Ct ^method. 

*Enzyme-linked immune sorbent assay for Caspase-3*

The concentrations of Caspase-3 level was determined on cell lysate of transfected/untransfected followed with sorafenib treated cellsfor 48 hrs comparing to negative control using ELISA kit (BioSource, CA, USA) according to the manufacturer’s instructions. All samples were assayed in triplicate and standard curve was plotted and used to calculate the concentrations.

*Cell cycle assay*

After 48 hrs treatment with sorafenib, the transfected/untransfected cells and negative control were harvested and washed twice with cold PBS. The cell suspension was adjusted to a concentration of 1 X 10^6^ cells per ml. Cells were fixed in 100 % ethanol at 4°C overnight then cells were resuspended in 200 µL 1X propidium iodide (PI), incubated at 37°C in the dark for 20 – 30 min placed on ice. G0 /G1, S and G2/M phases’ populations were detected by PI detector with flow cytometry (Beckman Coulter, USA) and analyzed by flowing software version 2.5.1(Turka centre of biotechnology, Turka uni, Finland).

*Cell apoptosis analysis*

Annexin V-FITC/PI (fluorescein isothiocyanate/propidium iodide) apoptosis detection kit (BestBio, Shanghai, People’s Republic of China) was used according to the manufacturer’s protocols for detection of apoptotic cells via flow cytometry in transfected and untransfected cells before and after sorafenib treatment comparing to negative control. Briefly, cells were collected and centrifuged for 10 min at 1,200 rpm. After washing with PBS, cell suspension was adjusted to a concentration of 1 X 10^6^ cells per ml and stained with 10 μL of Annexin V-FITC for 15 minutes and 5 μL of PI for 5 minutes at 4°C in the dark. Cells were then analyzed using FITC signal detector and PI detector with flow cytometry (Beckman Coulter, USA), and flowing software version 2.5.1 (Turka centre of biotechnology, Turka uni, Finland)

*Statistical analysis*

Statistical analyses were performed using GraphPad Prism version 7 for windows (GraphPad Software, CA, USA).Data are presented as mean ± standard deviation (SD). Multiple comparisons were carried out using one way analysis of variance (ANOVA) followed by Tukey test for post-hoc .Statistical significance was acceptable at a level of p-value ˂ 0.05. The two-tailed Student’s t test was used for all real time RT-PCR, cell cycle, apoptosis and caspase-3 level data. P-value threshold < 0.05 was considered significant. 

## Results

*Effect of sorafenib on cell proliferation*

Both cell lines used in the present study (HepG2/Huh7) demonstrated slightly difference in their sensitivity to sorafenib. HepG2 cells exhibited more sensitivity rather than Huh7 cells after treatment for 48 hrs. The calculated IC50 for HepG2 and Huh7 cells was 8 uM and 10 uM respectively. Based on this data, sub lethal doses of sorafenib (IC_20_ and IC_35_) were selected for treatment of cells in the subsequent experiments.

*Effect of combinedtreatment of miR-27a inhibitor/Sorafenib on proliferation rate of HepG2 and Huh7 cell lines*

**Figure 1:**
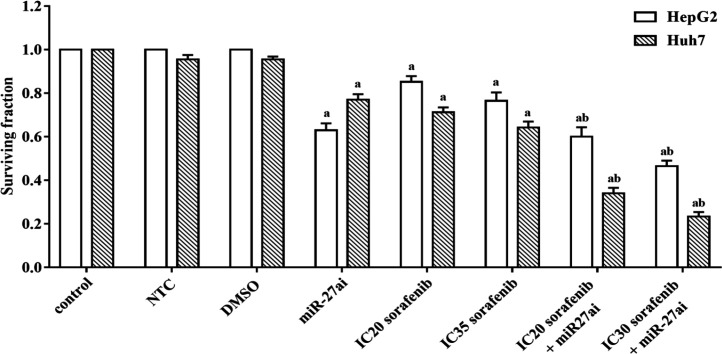
Effect of different doses of sorafenib (IC_20_ and IC_35_) on the surviving fraction of HepG2 and Huh7 cells transfected with 50nM miR-27a inhibitor for 48 hrs. The results are expressed as the mean±SD of 3 independent experiments. The statistical significance of the results was analyzed using one way ANOVA followed by Tukey multiple comparison test. a Significantly different from controls (control, NTC and DMSO), b from sorafenib only treated cells (p≤ 0.05).

Combining treatment of Anti-miR-27a and sorafenib was performed to determine their anti-tumor effect on HepG2 or Huh7 cells proliferation. A significant decreasing in proliferation was reported in both cell lines (HepG2 and Huh7) transfected with miR-27a inhibitor alone by 37% and 18% (Figure 1) compared with NTC (p < 0.05). While, combined treatment of miR-27a inhibitor with sorafenib (IC_20_) resulted in a significant inhibition in cell viability by 40% and 71% in HepG2 cells and Huh7 respectively compared to NTC (p < 0.05). The combined treatment of miR-27a inhibitor with higher dose of sorafenib (IC_35_) resulted in a higher significant inhibition in cell viability by 52.5% and 76.5% in Hepg2 cells and Huh7 respectively compared to NTC (p<0.05).Negative control and solvent (DMSO) did not show any significantly effect on cell viability for both cell lines. The data revealed that combining the antisense miR-27a with sorafenib could enhance the antitumor effect of sorafenib through declining the number of viable cells in both cell lines. Accordingly, we selected the best anti-proliferative effect of sorafenib (IC_35_) with miR-27ai for the analysis of cell cycle, apoptosis and caspase-3 assays. 

*Effects of Sorafenib or miR-27a inhibitor on Expression level of miR-27a in HepG2 and Huh7 cell lines*

**Figure 2:**
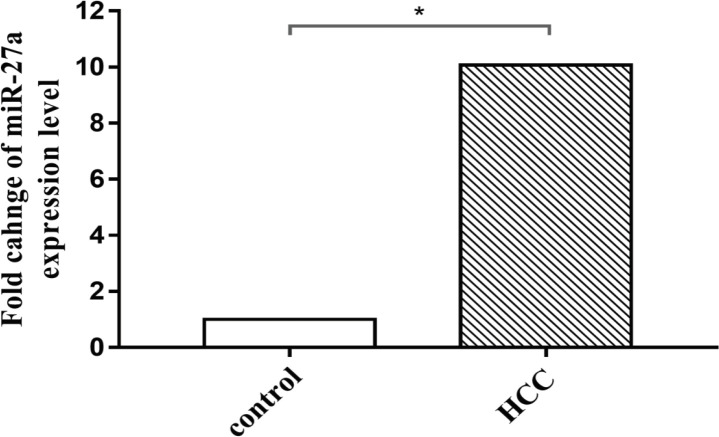
Fold Changes of miRNA-27a Expression Level Using qRT-PCR Assay in RNA Isolated from FFPE Tissue Sample of Primary Hepatocellular Carcinoma Patients Compared with Control Tissues from Healthy Volunteers. The results are expressed as the mean ± SD of 3 independent experiments. The statistical significance of the results was analyzed using the two-tailed Student’s t test (* p≤ 0.05)

Using quantitative RT-PCR assay, a significant up-regulation of miR-27a expression was reported in RNA isolated from FFPE specimens of 19 primary HCC patients analyzed compared with their corresponding healthy controls (Figure 2). The mean fold change recorded was about 10.072 (P<0.0206). This data confirmed overexpression of miR-27a in primary HCC tissues that refer to its oncogenic function.

**Figure 3:**
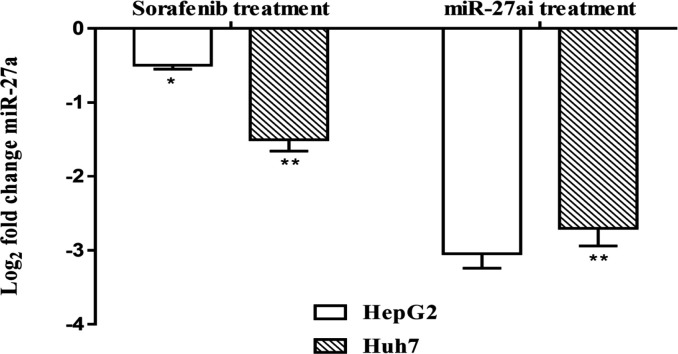
Gene Expression Analysis of miR-27a Using qRT-PCR Assay in Hepatocellular Carcinoma Cells, HepG2 and Huh7, after Treatment with Sorafenib or miR-27a Inhibitor Compared with Control Untreated Cells. Data were represented as mean values± SD for three independent experiments. The statistical significance of the results was analyzed using the two-tailed Student’s t test (* p≤ 0.05, ** p ≤ 0.01)

Moreover, we investigated whether sorafenib or miR-27a inhibitor influence the expression level of miR-27a in HepG2 and Huh-7 cell lines. As shown in Figure 3, a significant decrease in miR-27a expressionlevel was reported for both cell lines after treatment with sorafenib where log fold changes of miR-27a level were-0.5 and -1.5 for both cell lines respectively (P<0.01).Furthermore, highly significant down-regulated (-3.2 and -2.8) miRNA-27a expression was detected in both cell lines after transfection with miR-27ai indicating its successful transfection process (P<0.01). 

*Effect of combined treatment (miR-27ai and sorafenib) on cell cycle and apoptosis of HepG2 and Huh7 cell lines*

**Figure 4:**
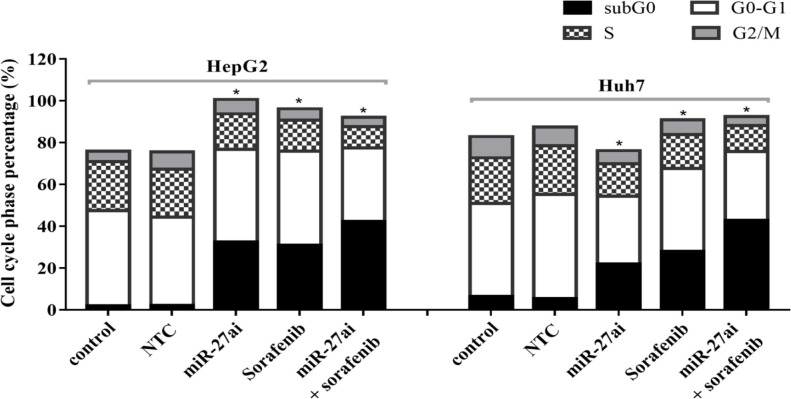
Flow Cytometry of Cell Cycle Analysis in HepG2 and Huh7 Cell Lines Using PI Staining. The percentage of each cell cycle for both cells lines in different treated groups was represented. The statistical significance of the results was analyzed using the two-tailed Student’s t test (* p≤ 0.05).

We investigated the effect of combination of anti-miR-27a and sorafenib (IC_35_) on cell cycle progression using flow cytometry for HepG2 and Huh7cell lines. A significant decreasing in the number of cells in S-phase was reported when cells treated with miR-27ai or sorafenib alone for both cell lines (p<0.05). Furthermore, a significant decreasing was recorded when cells exposed to combined treatment (miR-27ai+sorafenib).A significant increasing was also reported in number of cells in sub G0-phase for combined treated cells (p<0.05) compared with control untreated cells (Figure 4). 

**Figure 5:**
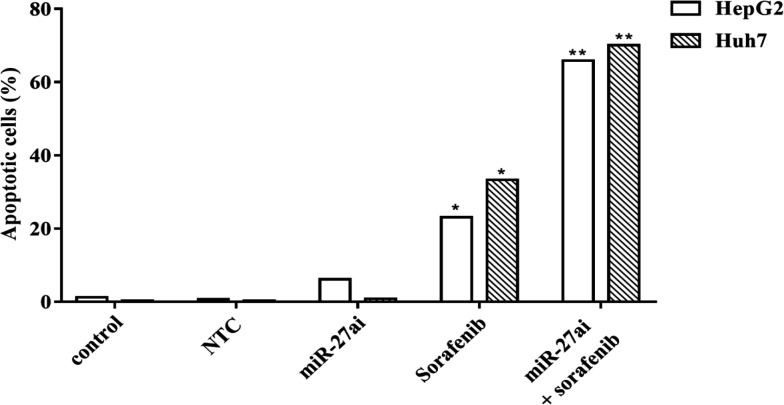
Flow Cytometry of Apoptosis Analysis in Hepg2 and Huh7 Annexin V-FITC and PI Staining Assay. Percentage of apoptosis was represented for both cells lines in different group analyzed. The statistical significance of the results was analyzed using the two-tailed Student’s t test (* p≤ 0.05)

In this study we next evaluated the apoptotic induction in HepG2 and Huh7 cells which co-treated with anti-miR-27a followed by sorafenib (IC_35_) via assessment the percentage of apoptotic cells by flow cytometry. Significant increase in apoptotic cell populations in HepG2 and Huh7 cells was observed when treated with sorafenib alone (p<0.05) and this rate demonstrated highly significant for cells transfected with miR-27a inhibitor followed by treatment with sorafenib compared with control cells (p<0.01). The percentage of apoptotic rates recorded for HepG2 transfected with miR-27ai, sorafenib alone, and combined treatment were about 6.15%, 23.1% and 65.9 versus control 1.21% respectively.Furthermore, the percentage of apoptotic rates for Huh7 cells recorded were 0.87%, 33.3% and 70.1% versus control 0.32% respectively as indicated in Figure 5.

**Figure 6:**
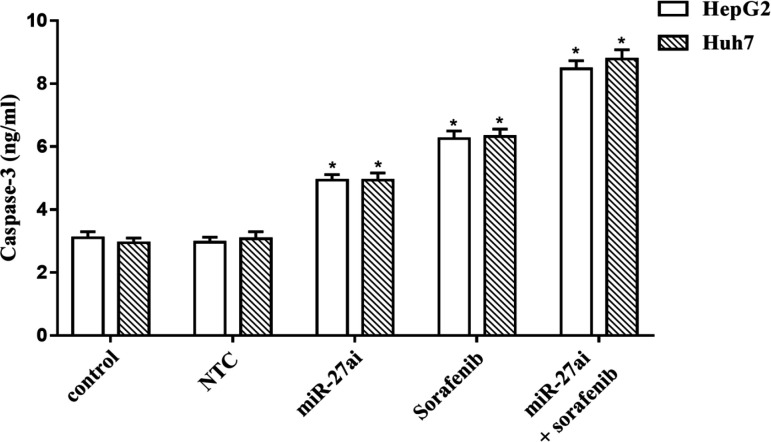
Caspase-3 Level (ng/ml) Hepatocellular Carcinoma Cells, HepG2 and Huh7, after Treatment with Sorafenib, miR-27a Inhibitor and Combination Compared with Control Untreated Cells Using Elisa Assay. Data were represented as mean values± SD for three independent experiments. The statistical significance of the results was analyzed using one way ANOVA followed by Tukey multiple comparison test. a Significantly different from controls (NC, NTC and solvent), b from sorafenib only treated cells (p≤0.05) considering significant

To further verify the anti-tumor effect of miR-27ai+ sorafenib induced cell apoptosis, we examinedthe level of caspase-3 activity, a hallmark of apoptosis, on both cell lines using Elisa assay. As shown in figure 6, there is a significant increasing in its level for miR-27ai transfected cells or sorafenib treated cells alone which became highly elevated for combination treated cells compared with either of control or single agent treatment (p<0.05). 

## Discussion

Therapeutic modulation of a single miRNA may affect many pathways simultaneously to achieve more clinical benefit (Ebert et al., 2007). Hence targeting aberrantly expressed miRNA is logical and attractive. The widespread overexpression of miR-27a in cancer has led to the belief that miR-27a is an oncogenic microRNA. Cell culture and animal experiments supported this speculation, showing that the down-regulation of miR-27a expression can suppress cell proliferation and slow tumor growth. Some reports demonstrated the oncogenic role of miR-27a which plays important role in the development of HCC and might thus be a new therapeutic and prognostic target in HCC patients (Li et al., 2015). 

Several recent studies confirmed the protooncogenic characteristic of miR-27a in different types of cancer. It has been shown that overexpression of miR-27a was associated with high cell proliferation and metastasis in gastric cancer cells through mediating suppression of PH domain and leucine-rich repeat protein phosphatase 2 (PHLPP2) leading to stimulation of the AKT/GSK3B pathway (Ding et al., 2017). Moreover, overexpression of miR-27a enhanced tumor metastasis by stimulating epithelial-mesenchymal transition process in breast cancer which added further evidences for its potential application in cancer therapy (Jiang et al., 2018). High expression of miR-27a level detected intissues ofprimary HCC patients in the current data are consistant with that previously reported studies confirming its oncogeneic role in HCC. 

The discovery of targeted therapy with multi-tyrosine kinase inhibitors, such as sorafenib has set abreakthrough for patients with advanced cancer. Its antitumor mechanisminvolving the inhibiton mostly known pathways associated with hepatocarcinogenesis include the Ras/Raf/MAP/ERK, the PI3K/Akt/mTOR, the Wnt/β-catenin and the JAK/STAT pathways (Wilhelm et al., 2005) but its mechanism of action still has not been fully elucidated. Emmerging evidences considering miRNAs as important key players in HCC pathogenesis. Numerous experimental and clinical studies revealed that the aberrant expression of miRNAs is associated with the progression of hepatocellular carcinoma (Callegari et al., 2013; Lu et al., 2005). Moreover, various studies highlighted changes in miRNA expression profiles in response to sorafenib and other therapeutics (Peveling-Oberhag et al., 2015; Stiuso et al., 2015). A study done on HCC cells demonstrated that miR-142-3p sensitised cells to sorafenib by promoting apoptosis and inhibiting cell growth and proliferation via targeting autophagy related 5 (ATG5) and autophagy-related 16-like 1 (ATG16L1) which are the main targets of autophagy regulating miR-142-3p (Zhang et al., 2018). Delcining of miR-221 overexpression level using its inhibitor modulate sensitized HCC cells via induction of caspase-3, a pro-apoptotic target of miR-221 (Fornari et al., 2017). It has been indicated recently that miR-140-3p can enhance the antitumor effect of sorafenib via targeting pregnane X recptor (PXR) resulting down-regulation of drug-resistance related genes in HCC cells (Li et al., 2018).

We explored the feasibility of regulation of miR-27a using chemically modified anti-miR Oligos alone and in combination of Sorafenib in HCC cells. Our data showed that combining of antisense miR-27a Oligos with sorafenib sensitized HepG2 and Huh7 to sorafenib-induced cell death compared to sorafenib-onlytreated cells and controls, indicating that antisense miR-27acould potenitiate the antitumor effect of sorafenib through declining the number of viable HCC cells in vitro. Previous study demonstrated using a lentiviral vector to stably expression of anit-miR-27a targeting the oncogenic miRNA declines proliferation of glioma cell line suggesting its potential role for suppression of malignancy (Feng et al., 2012). Xu et al., (2013) proposed that inhibtion of miR-27a by its antisense oligoenhanced the response of ovarian cancer cell line SKOV3 to Genistein and decrease cell growth and metastasiss by upregulating the Sprouty2, the putative miR-27a target gene. 

An up-regulation of miR-27a was observed significantly in HCC tissues and different cell lines. Furthermore, its down-regulation using miR-27a inhibitor declined proliferation, induced apoptosis and blocked the G1/S cell cylce transition via targeting the 3’-untranslated region of peroxisome proliferator-activated receptor γ (PPARγ) using dual luciferase assay (Li et al., 2015). In gastric cancer cells, the reduction of miR-27a inhibited cell growth in both in vitro and nude mice assays which referred to its important role in cell proliferation (Zhao et al., 2011). Moreover, the most exciting finding in the present study was observed where a significantdeclining of cell count in S-phase of combined treated cells compared with either sorafenib or miR-27a inhibitor alone as shown by cell cycle analysis. Previous study done on the same cell lines reported reduction of cell counts in S phase upon treatment with anti-miR-27a olignonucleotides (Li et al., 2015) 

Apoptosis is the one of dysfunctioning signaling pathways in HCC. The effects of administration of sorafenib on inducing apoptosis were investigated in many studies (Garten et al., 2019; Bahman et al., 2018). 

In the current work, apoptosisanalysis conducted by flow cytometry demonstrated a sigificant increase in apoptotic rate in cells treated with combination therapy compared with cells treated with either sorafenib or miR-27a inhibitor alone. Finding of Li et al., (2018) demonstrated a significant increase in apoptotic rate in HepG2 transfected with miR-27a inhibitor whichverifiedthe additionnal effect of combinational therapy (antisense miR-27a and sorafenib) on apoptotic rate of hepatocellular carcinoma cells.

To gain insight the molecular mechanisms in miR-27a mediated apoptosis ,caspase-3 level was assesed in HepG2and Huh7 transfected cells withantisense miR-27a. The results showed a significant increase in the caspase-3 in both transfected cells compared to controls. Remarkably trasfected cells which co-treated with sorafenib demonstrated an enhancement in caspase-3 level compared to treatmet with either sorafenib or miR-27a alone. The elevated level of the assessed caspase-3 upon transient ransfection of miR-27a inhibitorin HepG2 and Huh7 might proposed to the negative correlation between miR-27a and APAF-1, a key mediator in cytochrome C-dependent apoptotic pathway (Zhang et al., 2017). The author considered APAF-1 is the direct target of miR-27a.

Our finding data demonstrated that blocking miR-27a plays a crucial role in cell proliferation, cell cylce and apoptosis process in hepatocellular carcinoma cell lines.Furthermore, the antitumor effect of miR-27a inhibitor in combination with sorafenib on hepatocellular cell lines might alter its therapeutic strategy. 

## Author Contribution Statement

AA, conceived, designed and supervised the study. EA, performed the experiments and analyzed the data. AA, shared in study design and technical guidance of study. AA, AA, MA, and HS, analyzing and interpretation of data as well as drafting the manuscript. All authors read and approved the final manuscript. 
